# Intrapartum Diagnosis and Treatment of Longitudinal Vaginal Septum

**DOI:** 10.1155/2014/108973

**Published:** 2014-05-07

**Authors:** Antonio Henriques de França Neto, Bianca Virgolino Nóbrega, Jessé Clementino Filho, Tiago Cavalcanti do Ó, Melania Maria Ramos de Amorim

**Affiliations:** ^1^The Medical Residency Program in Obstetrics and Gynecology at the Federal University of Campina Grande (UFCG), Rua Antônio de Souza Lopes 120, Catolé, Apartamento 1204, 58410-180 Campina Grande, PB, Brazil; ^2^Gynecology, School of Medical Sciences, Rua Antônio de Souza Lopes 120, Catolé, Apartamento 1204, 58410-180 Campina Grande, PB, Brazil; ^3^Obstetrics and Gynecology, UFCG, 58410-180 Campina Grande, PB, Brazil; ^4^Programa de Valorização do Profissional da Atenção Básica (PROVAB), 58410-180 Campina Grande, PB, Brazil

## Abstract

Longitudinal vaginal septum is a rare Müllerian malformation that may be associated with dyspareunia, dysmenorrhea, primary amenorrhea, and infertility. In this report, the authors present a case of longitudinal vaginal septum in a 15-year-old patient with a full-term pregnancy whose diagnosis was only made during labor following bidigital vaginal and speculum examination. Septoplasty was performed during the second stage of labor. Both mother and child progressed satisfactorily and were discharged from hospital in good health. Six months later, ultrasonography, hysterosalpingography, and hysteroscopy were carried out and no other associated abnormality was found.

## 1. Introduction

The complex development of the genital tract during embryogenesis involves a series of events that include cell differentiation, migration, fusion, and canalization [1]. Failure at any stage in this process may result in a congenital abnormality.

The American Society for Reproductive Medicine classifies abnormalities of the female genital tract into six separate categories based on their clinical presentation and on fetal prognosis after treatment [2]. This classification does not include nonuterine abnormalities but permits additional descriptions for associated vaginal, tubal, and urinary anomalies.

The proximal two-thirds of the vagina are formed from the fusion of the Müllerian ducts, while the distal third originates from the urogenital sinus [1]. The sinovaginal bulbs, two solid evaginations originating in the urogenital sinus at the distal extremity of the Müllerian tubercle, proliferate at the caudal end of the uterovaginal canal to become a solid vaginal plate. The lumen of the lower vagina is then formed via apoptosis of the central cells in this vaginal plate, extending in a cephalic direction. Complete canalization occurs by 20 weeks of intrauterine life [1]. On the other hand, the Müllerian ducts fuse together between the 11th and 13th weeks of intrauterine life, with this fusion and subsequent absorption occurring in a caudal-cranial direction [3–6].

In recent decades, advances in imaging techniques have facilitated diagnosis of Müllerian duct anomalies, the incidence of which is estimated at 0.001 to 10% [3]. One of these abnormalities is transverse vaginal septum, a vertical fusion disorder between the Müllerian ducts and the urogenital sinus, which has been linked to autosomal recessive transmission [4]. It is unusual for pregnancy to occur when these malformations are present, since many of them may cause infertility.

Longitudinal vaginal septum is typically associated with uterine anomalies such as a septate uterus or uterus didelphys [7]. The septum that divides the vagina may be partial or complete. It may present clinically as a difficulty in inserting tampons, persistent bleeding despite the presence of a tampon, or dyspareunia. On the other hand, it may be asymptomatic.

Treatment involves complete removal of the septum. Excision is the traditional procedure, with care being taken to avoid causing bladder or rectal lesions. The tissue should be completely excised, since remaining fragments of the septum may cause dyspareunia. The septal tissue is resected and the normal vaginal mucosa on each vaginal wall is sutured together along the length of the defect made by the resection. Surgery is not necessary in asymptomatic women with a longitudinal vaginal septum; however, carrying out the procedure will certainly facilitate a subsequent vaginal delivery. To the best of our knowledge, no previous reports have been published on the resection of longitudinal vaginal septum during labor.

## 2. Case Presentation

NFB, a 15-year-old girl, gravida 1, para 0, was admitted to the teaching hospital of the* Fundação Assistencial da Paraíba* (FAP) on September 13, 2011, at 37 weeks of gestation according to the date of her last menstruation, which was corroborated by a first-trimester ultrasonography scan. She reported having had intermittent lower abdominal pain for the preceding two hours.

**Figure 1:**
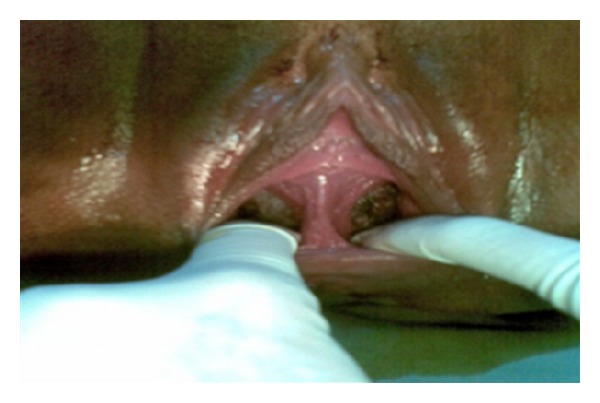
Longitudinal vaginal septum detected during delivery.

Physical examination revealed the patient to be in good general health, with a normal complexion, well-hydrated, afebrile, acyanotic, anicteric, with no edema, and alert and oriented to place and time. Her blood pressure was 110 × 70 mmHg, heart rate 84 bpm, respiratory rate 18 breaths per minute, and cardiopulmonary auscultation normal. The patient had a gravid abdomen; fundal height 36 cm; fetus in cephalic presentation. Fetal heart rate (FHR) was 140 bpm, monitored in the lower left quadrant, with three contractions of 30 seconds each in ten minutes. Digital vaginal examination revealed a cervix dilated to 4 cm, cephalic presentation, Hodge 1 (i.e., at the level of the pelvic inlet), left occiput anterior (LOA) position, and membranes still intact. An elastic structure around 3 cm behind the vaginal introitus was palpable. It was painless to the touch and extended from the anterior wall to the posterior wall of the vagina. A more detailed examination was made by inserting a Collins speculum, revealing a longitudinal vaginal septum in the distal third of the vagina (Figure 1).

Expectant management was preferred in view of the patient's good obstetric conditions. Labor progressed satisfactorily and the second stage occurred around eight hours after admission.

**Figure 2:**
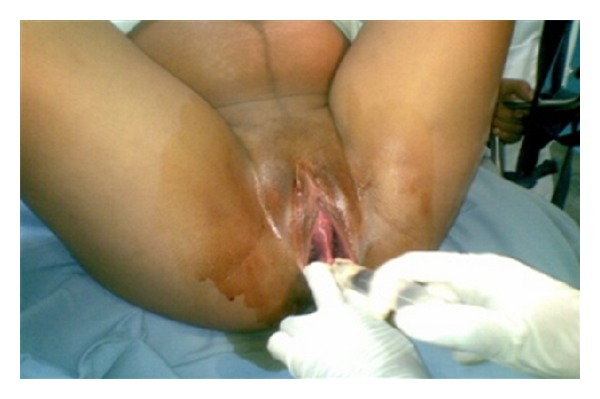
Anesthetic injection into the septum.

**Figure 3:**
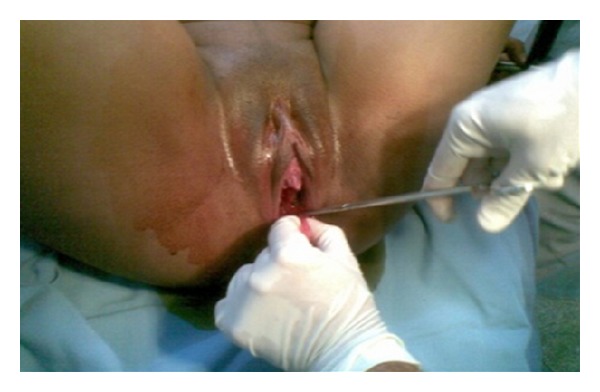
Intrapartum resection of the septum performed using scissors.

**Figure 4:**
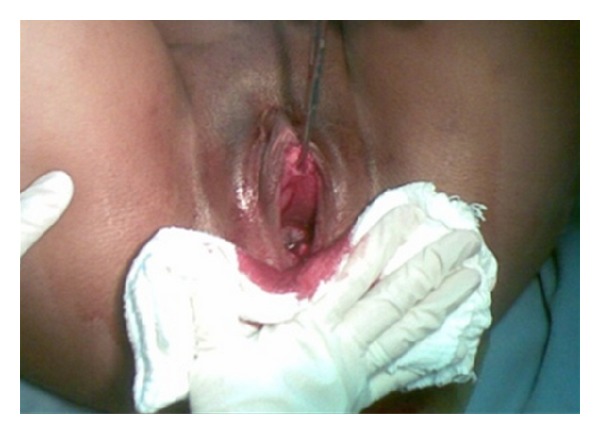
Delivery following resection of the septum. Note the anterior extremity of the septum is still clamped (sutures performed following delivery).

During the second stage of labor, with the pregnant woman in a semiseated position on the delivery bed and the fetal head already in the vaginal canal, the septum was clamped and resected, permitting the birth of a male infant weighing 3600 grams and measuring 51 cm. Apgar scores were 9 at the first minute and 10 at the fifth minute. No deformities or malformations were apparent. Childbirth occurred without complication. Episiotomy was not required. Prophylactic treatment with an intramuscular injection of 10 IU of oxytocin was given to prevent postpartum hemorrhage. After delivery of the placenta, the other extremity of the septum was clamped and subsequently resected, completely restoring the anatomy of the genital tract. No lacerations were detected (Figures 2, 3, and 4). Next, both resected bases were sutured with simple continuous sutures using plain catgut 2-0, and the patient was released to return to the ward.

The postpartum was uncomplicated and the patient was discharged on September 15, 2011. She was followed up at the gynecology clinic of the Federal University of Campina Grande and six months after delivery she was submitted to three-dimensional ultrasound, hysterosalpingography, and hysteroscopy for reevaluation. No associated abnormalities were found. She was initially prescribed progestin-only oral contraceptives; however, after she had stopped breastfeeding her prescription was changed to a combined estrogen/progesterone pill. She has not expressed any desire to become pregnant again up to the present date. A further gynecological examination was normal except for the scars on the anterior and posterior vaginal walls resulting from the septum resection.

## 3. Discussion

Although a rare condition, longitudinal vaginal septum should always be taken into consideration in differential diagnoses when a varying combination of dyspareunia, cyclic pelvic pain, hematocolpos, hematometra, and mucocolpos is present, either associated or not with primary amenorrhea, which may be present when there is complete obstruction of the vaginal canal. Diagnosis and treatment should be timely in order to avoid possible complications such as pelvic adhesions and damage to the fallopian tubes, principally in cases of complete obstruction, as well as the discomfort and psychological repercussions of painful symptoms such as dyspareunia [2, 3]. The principal differential diagnoses are vaginal agenesia and imperforate hymen [3, 4]; possibilities that were eliminated in the present case due to the obvious fact that they would have prevented fertilization and pregnancy.

An important aspect of the present case lies in the fact that the vaginal septum was located posteriorly, around three centimeters from the vaginal introitus, which makes it unusual, with only 15–20% of all cases occurring at this site compared to approximately 46% located anteriorly in the vagina [2, 3]. It should also be emphasized that there were no symptoms prior to diagnosis, with the septum only being identified during labor, an even more unusual situation.

The relative delay in diagnosing this anatomical abnormality may be justified by the fact that in this case the vaginal septum was partial. This fact helped attenuate symptoms of dyspareunia and dysmenorrhea in addition to permitting menstrual flow, whereas the presence of primary amenorrhea could have caused severe problems in puberty [3, 6].

The lack of symptoms may have contributed to the fact that the condition was not diagnosed during the patient's prenatal care. This raises the question, however, of why she had never been submitted to a vaginal examination during her prenatal care. If a speculum had ever been inserted, the septum would certainly have been seen and its excision would have been scheduled prior to delivery. On the other hand, in our opinion, the fact that diagnosis was not made during prenatal care ultimately avoided a possible (and unnecessary) Cesarean section.

The principal tools for diagnosing longitudinal vaginal septum are ultrasonography, magnetic resonance imaging (MRI), and hysterosalpingography. These tests are recommended because they enable the thickness and site of the septum to be established in addition to alerting to the coexistence of other associated congenital defects [2, 3, 6]. In rare cases, ultrasonography can allow diagnosis to be made* in utero* during the third trimester of pregnancy, with the discovery of a cystic tumor of the pelvis in a female fetus [3].

In the present case, another relevant aspect is the fact that diagnosis was made exclusively on the basis of the physical examination, without the use of any of the above-mentioned supplementary tests, which were only requested following delivery. In thesis, this may be justified by the fact that the septum in question was situated low down in the vagina, allowing visualization in great detail and enabling the precise thickness to be determined without further investigation. In addition, it was possible to visualize the rest of the vaginal canal and cervix, thus eliminating the possibility of one of the principal differential diagnoses of this condition, which is congenital absence of the cervix.

Longitudinal vaginal septum may lead to fertility problems [6]. In the case described here, this dysfunction was notably absent, since the patient, still in adolescence, was already pregnant and had conceived without the benefit of any assisted reproductive techniques.

Publications in both national and international literature recommend surgery in all cases of LVS and also suggest that resection must be performed early in cases of complete septum. Excision is the procedure of choice for the treatment of longitudinal vaginal septum; however, care should be taken not to provoke any accidental lesion to the rectum or bladder. Although it is not obligatory in asymptomatic women with LVS, surgery should be performed whenever the woman wishes to become pregnant in order to facilitate a subsequent normal delivery. In addition to the traditional technique described above, there are reports in the literature of alternative procedures. Good results have been reported with hysteroscopic resection, a technique that is used principally in young patients to permit preservation of the hymen [8].

In addition to the fact that diagnosis was only made intrapartum, labor was allowed to progress normally in the present case, with the septum being resected during the second stage of labor. In fact, resection of a longitudinal vaginal septum may be performed during vaginal delivery, thus avoiding a Cesarean section, which would certainly increase the likelihood of morbidity in the patient. Ideally, resection should be performed prior to the patient becoming pregnant or early in pregnancy; however, since the septum was relatively thin in the present case, resection was simple and more complex techniques were not required. Considering the greater maternal and perinatal morbidity and mortality associated with Cesarean sections [9, 10], waiting for labor to begin is justified, since the septum can be resected during the course of labor or during delivery, depending on the characteristics of each individual case.

To the best of our knowledge, the case described here is the only report of a partial longitudinal vaginal septum managed during delivery, making it unique and justifying its publication. Furthermore, the efficacy of the form of management adopted here is endorsed by the patient's excellent postoperative conditions and the normal delivery of a healthy infant.
